# Association analyses confirm five susceptibility loci for systemic lupus erythematosus in the Han Chinese population

**DOI:** 10.1186/s13075-015-0602-9

**Published:** 2015-03-28

**Authors:** Yu-jun Sheng, Jian-hua Xu, Yong-gui Wu, Xian-bo Zuo, Jin-ping Gao, Yan Lin, Zheng-wei Zhu, Lei-lei Wen, Chao Yang, Lu Liu, Yu-yan Cheng, Yan Chang, Lu-lu Yang, Fu-sheng Zhou, Xian-fa Tang, Xiao-dong Zheng, Xian-yong Yin, Hua-yang Tang, Liang-dan Sun, Yong Cui, Sen Yang, Xue-jun Zhang

**Affiliations:** Institute of Dermatology and Department of Dermatology, No.1 Hospital, Anhui Medical University, 81 Meishan Road, Hefei, Anhui 230032 China; Key Laboratory of Dermatology, Anhui Medical University, Ministry of Education, China, Hefei, Anhui 230032 China; State Key Laboratory Incubation Base of Dermatology, Anhui Medical University, Hefei, Anhui 230032 China; Collaborative Innovation Center for Complex and Severe Dermatosis, Anhui Medical University, Hefei, Anhui 230032 China; Department of Rheumatology and Immunology, No.1 Hospital, Anhui Medical University, Hefei, Anhui 230032 China; Department of Nephrology, No.1 Hospital, Anhui Medical University, Hefei, Anhui 230032 China; Department of Dermatology, China-Japan Friendship Hospital, Beijing, 100029 China

## Abstract

**Introduction:**

Systemic lupus erythematosus (SLE) is a multisystem autoimmune disease. Currently, numerous genetic loci of SLE have been confirmed. Here we try to further explore additional genes contributing to SLE susceptibility in this study.

**Methods:**

Forty nine single nucleotide polymorphisms (SNPs) with moderate-risk for SLE in previous study were genotyped in a large-scale replication study with a total of 3,522 cases and 8,252 controls using the Sequenom Massarray system. Association analyses were performed using logistic regression with gender or sample cohorts as a covariate through PLINK 1.07 software.

**Results:**

This replication effort confirmed five reported SLE susceptibility loci reaching genome-wide levels of significance (*P*_*meta*_ <5.00 × 10^−08^): *TNFSF4* (rs1418190, odds ratio (OR) = 0.81, *P*_meta_ = 1.08 × 10^−08^; rs4916219, OR = 0.80, *P*_meta_ = 7.77 × 10^−09^), *IRF8* (rs2934498, OR = 1.25, *P*_meta_ = 4.97 × 10^−09^), *miR-146a* (rs2431697, OR = 0.69, *P*_meta_ = 1.15 × 10^−22^), *CD44* (rs2732547, OR = 0.82, *P*_meta_ = 1.55 × 10^−11^), and *TMEM39A* (rs12494314, OR = 0.84, *P*_meta_ = 1.01 × 10^−09^). Further logistic regression analysis indicated that the genetic effects within *TNFSF4* detected in this study are independent from our previously reported signals.

**Conclusions:**

This study increases the number of established susceptibility loci for SLE in Han Chinese population and highlights the contribution of multiple variants of modest effect. Although further studies will be required to identify the causal alleles within these loci, the findings make a significant step forward in our understanding of the genetic contribution to SLE in Chinese population.

**Electronic supplementary material:**

The online version of this article (doi:10.1186/s13075-015-0602-9) contains supplementary material, which is available to authorized users.

## Introduction

Systemic lupus erythematosus (SLE) is an important model of autoimmunity characterized by the presence of antibodies to nuclear self-antigens and involvement of multiple organs. The etiopathogenesis of SLE is complex and still largely unknown. Both genetic and environmental factors contribute to disease susceptibility [1]. The prevalence of SLE and its manifestations varies between different ethnic and geographical populations [2], with higher prevalence rates and more severe complications in non-European populations. For example, higher rates of lupus nephritis have been observed in Asians [3,4], Hispanics [5], and African Americans [5,6]. In contrast, higher prevalence rates of photosensitivity have been noted in Europeans [7]. The ethnic and genetic heterogeneity of SLE may contribute to these differences in SLE manifestation rates.

Despite considerable clinical heterogeneity, SLE is one of the most heritable autoimmune diseases, with a sibling risk ratio of around 30 [8]. Increased understanding of the underlying genetic basis for SLE is of key importance in improving the prognosis of patients with SLE. Recent genome-wide association studies (GWAS) and candidate gene studies have confirmed genetic associations of over 40 loci with SLE risk that achieve genome-wide significance (*P* <5 × 10^−8^) [9]. Clustering of some genetic associations identified to date appears to fall into at least three major pathways, including type I interferon (IFN) and NF-κB pathways, lymphocyte signaling, and immune complex processing [10]. All of these pathways are of potential importance in the pathogenesis of SLE. However, these genetic risk loci cannot fully explain the genetic susceptibility to SLE and some clinical features, suggesting additional genetic factors yet to be discovered. To detect more additional SLE risk loci, we selected 49 single nucleotide polymorphisms (SNPs) from 40 distinct loci that showed nominal evidence of association to SLE (*P* <0.01) in our genome-wide dataset [11] and followed up two replications in two large independent sample cohorts of Han Chinese.

## Materials and methods

### Sample collection

A total of 4,556 SLE patients and 9,451 controls recruited from multiple cooperation hospitals in China were included in this study. The sample information is summarized in Table 1.We included original data from the 1,047 cases and 1,205 controls in the initial stage [11] and the replication samples consisted of two independent cohorts: replication 1 (2,202 SLE cases and 2,208 healthy controls) and replication 2 (1,307 cases and 6,038 healthy controls) from the Han Chinese population. All samples were of self-described Han Chinese descent and cases were confirmed as having SLE by using the revised criteria for the classification of SLE from the American College of Rheumatology (ACR) [12]. Clinical data were collected at each hospital from the affected individuals through a full clinical checkup by at least two physician specialists. Additional demographic information was collected from both cases and controls through a structured questionnaire. The controls’ samples were clinically assessed to be without SLE, other autoimmune disorders, systemic disorders or family history of autoimmune disorders (including first-, second- and third-degree relatives). The Institutional Ethical Committee of The First Affiliated Hospital of Anhui Medical University, according to Declaration of Helsinki principles, approved this study, and all participants provided written informed consent.

**Table 1:** **Summary information of samples used in genome-wide association studies (GWA**S) **and replication studies**

	**Cases**					**Controls**			
	**Sample size**	**Mean age (SD)**	**Mean age of onset (SD)**	**Male/female**	**Female, %**	**Sample size**	**Mean age (SD)**	**Male/female**	**Female, %**
GWAS	1,047	34.02 (11.53)	29.81 (10.08)	63/984	93.98	1,205	34.75 (12.97)	673/532	44.15
Replication 1	2,202	34.71 (11.91)	30.49 (11.20)	187/2,015	91.51	2,208	30.41 (13.00)	995/1,213	54.94
Replication 2	1,307	33.11 (11.63)	28.19 (10.65)	87/1,220	93.34	6,038	28.36 (10.92)	2,924/3,114	51.57
Total	4,556	34.10 (11.76)	29.70 (11.05)	337/4,219	92.60	9,451	29.65 (12.05)	4,592/4,859	51.41

EDTA anti-coagulated venous blood samples were collected from all participants in the study. We isolated genomic DNA from peripheral blood lymphocytes by standard procedures using Flexi Gene DNA kits (QIAGEN GmbH, Hilden, Germany). DNA was normalized to working concentrations of 50 ng/μl for genome-wide genotyping and 15 to 20 ng/μl for the validation study.

### Genotyping and quality control

Genotyping of the whole genomic was performed using Illumina Human 610-Quad BeadChips (Illumina, Inc., San Diego, CA, USA) in the State Key Lab Incubation of Dermatology, Ministry of Science and Technology, Anhui Medical University (AHMU). We excluded SNPs that had a low call rate (<98%), deviated from Hardy-Weinberg equilibrium (HWE) (*P*_HWE_ <0.0001 in controls), or the minor allele frequency (MAF) was less than 5% both in cases and controls. The validation study was conducted using the Sequenom Massarray system (Sequenom, Inc., San Diego, CA, USA). Allele detection was performed using MALDI-TOF mass spectroscopy. The mass spectrograms were analyzed by the MassARRAY TYPER software (Sequenom, Inc.). All of these experiments were performed according to the manufacturers’ protocols. Quality control was conducted on each dataset separately using PLINK 1.07 software [13]. According to quality-control measures, we excluded samples with call rates <90% and SNPs with call rates <95%, MAF (<5%) or HWE in controls with *P* <0.01. In total, 2,202 cases, 2,208 controls and 45 SNPs for replication 1, and 1,307 cases, 6,038 controls and 12 SNPs for replication 2 were available for each replication analysis.

### SNPs selection for replication

To search for additional variants associated with SLE, we selected 199 SNPs for further evaluation from our genome-wide dataset after excluding previously reported SLE risk alleles in our studies [11,14,15]. All of these selected SNPs met the following quality criteria: 1) the MAF was higher than 5% both in cases and controls; 2) HWE in controls with *P* ≥0.01 and HWE in cases with *P* >0.0001; 3) SNPs with *P* <0.01 after adjustment by gender; 4) proximity to putative candidate genes (immune-related or involved in immune cell proliferation and differentiation) or known susceptibility loci for autoimmune diseases; and 5) SNPs that appeared to have better evidence of association after further analysis through increasing the sample by another 489 controls. These controls were newly genotyped in a series of GWAS of various diseases in the Han Chinese population, including psoriasis [16], vitiligo [17], leprosy [18], atopic dermatitis [19], esophageal squamous cell carcinoma [20], and so on. However, these additional controls were not well-marched with cases. Therefore, these 489 controls were not included in the association analysis of the validation study or in the final joint association analysis of the combined samples. In each locus, one or two of the most significant SNPs were selected for the validation study. After pruning, 49 SNPs were selected for the replication 1.

### Statistical analysis

In the initial stage, we performed single-marker association analyses by logistic regression with gender and age as covariates in the GWAS dataset. Quality control has been described above. For the validation studies, HWE tests in controls were calculated using PLINK 1.07 software. We further excluded SNPs with a call rate of <95% in cases or controls and with HWE in the controls (*P* <0.001). Additionally, cluster patterns of the genotyping data from the Illumina and Sequenom analyses were checked to confirm their quality. We performed heterogeneity tests (*I*^*2*^ and *P-*values of the *Q* statistics) among these three independent cohorts using the method described previously [21] and the extent of heterogeneity was assessed by using the *I*^*2*^ index. [22] The fixed effect model (Mantel-Haenszel) was when *I*^*2*^ was <30% that was considered as no heterogeneity [23]. Otherwise, the random effect model (DerSimonian-Laird) was implemented [24]. The joint analysis of all combined samples was performed using logistic regression with gender, age and sample cohorts as covariates. Gene annotations were adapted from the University of California at Santa Cruz Genome Browser [25].

## Results

### Association results of GWAS, replication 1 and combined samples of these two stages

In the replication 1, 49 SNPs were genotyped in 2,202 individuals with SLE and 2,208 healthy controls. After pruning, 45 SNPs that passed quality control were included for final analysis (Table 2 and Additional file 1: Table S1). The association evidence for 15 SNPs with SLE was replicated independently in the replication 1 (*P* <0.05). When the genotypic data from the GWAS and the replication 1 were combined, we identified two SLE susceptibility genes reaching genome-wide levels of significance (*P*_meta_ = 5.00 × 10^−08^): *TNFSF4* (rs1418190, odds ratio (OR) = 0.81, *P*_meta_ = 1.08 × 10^−08^; rs4916219, OR = 0.80, *P*_meta_ = 7.77 × 10^−09^), and *IRF8* (rs2934498, OR = 1.25, *P*_meta_ = 4.97 × 10^−9^) and another 12 SNPs also showed evidence suggestive of association (Table 2). Our data suggest that the association is independent from our previously reported signals in the neighboring SNPs (rs1234315 and rs2205960) [11], because we found no evidence of strong LD among them (D’ <0.22, *r*^2^ <0.02). To confirm that the two genetic effects detected are independent, we performed logistic regression analysis among them based on the GWAS data, tagged by rs1418190 or rs4916219 and rs1234315 or rs2205960, and showed that each genetic association remained significant (*P*_*condition*_ <8.42 × 10^−4^) after controlling for the effect of each of the remaining two SNPs, using an additive model (Additional file 2: Table S2). In addition, these two novel independent SNPs were confirmed in the haplotype association analysis (Additional file 3: Table S3).

**Table 2:** **Association evidence for 15 single nucleotide polymorphisms (SNPs) in genome-wide association studies (GWAS), replication and combined studies**

**Chr**	**SNP**	**Gene**	Stages*	**Allele**	**MAF**		**OR (95% CI)**	***P*** **-value** ^**a**^	**Meta-analysis**		***P***_***Q***_	***I*** ^**2**^
					**Cases**	**Controls**			**OR (95% CI)**	***P*** **-value** ^**b**^		
1q25	rs1418190	*TNFSF4*	i	G/A	0.3089	0.3579	0.80 (0.71, 0.91)	5.26 × 10^−04^	0.81 (0.75, 0.87)	1.08 × 10^−08^	0.92	0
			ii	G/A	0.3250	0.3738	0.81 (0.74, 0.88)	5.30 × 10^−06^				
1q25	rs4916219	*TNFSF4*	i	A/G	0.2722	0.3166	0.81 (0.71, 0.92)	1.15 × 10^−03^	0.80 (0.75, 0.86)	7.77 × 10^−09^	0.87	0
			ii	A/G	0.2903	0.3402	0.79 (0.73, 0.87)	1.74 × 10^−06^				
2p16.3	rs2048979	*NRXN1*	i	G/A	0.4173	0.4743	0.79 (0.71, 0.89)	1.26 × 10^−04^	0.87 (0.82, 0.91)	1.35 × 10^−03^	0.11	60.01
			ii	G/A	0.4238	0.4521	0.89 (0.82, 0.97)	7.72 × 10^−03^				
			iii	G/A	0.4146	0.4517	0.86 (0.79, 0.94)	6.14 × 10^−07^				
3q13.33	rs12494314	*TMEM39A*	i	G/A	0.2930	0.3318	0.83 (0.74, 0.95)	5.16 × 10^−03^	0.84 (0.80, 0.89)	1.01 × 10^−09^	0.87	0
			ii	G/A	0.2999	0.3455	0.81 (0.74, 0.89)	5.62 × 10^−06^				
			iii	G/A	0.2997	0.3363	0.84 (0.78, 0.94)	6.01 × 10^−08^				
3q13.33	rs6804441	*CD80*	i	G/A	0.2949	0.3436	0.80 (0.70, 0.91)	4.90 × 10^−04^	0.86 (0.82, 0.91)	5.90 × 10^−04^	0.17	46.88
			ii	G/A	0.3038	0.3394	0.85 (0.78, 0.93)	3.71 × 10^−04^				
			iii	G/A	0.3189	0.3359	0.93 (0.84, 1.02)	4.95 × 10^−02^				
3q13.33	rs2222631	*CD80*	i	G/A	0.4280	0.4743	0.83 (0.74, 0.93)	1.90 × 10^−03^	0.92 (0.88, 0.97)	9.08 × 10^−02^	0.05	74.41
			ii	G/A	0.4420	0.4666	0.91 (0.83, 0.99)	2.21 × 10^−02^				
			iii	G/A	0.4600	0.4605	1.00 (0.92, 1.09)	3.30 × 10^−01^				
3q29	rs9866504	*FLJ25996*	i	A/G	0.1148	0.1568	0.70 (0.59, 0.83)	4.43 × 10^−05^	0.85 (0.79, 0.92)	6.44 × 10^−02^	0.01	83.81
			ii	A/G	0.1248	0.1471	0.83 (0.73, 0.94)	2.34 × 10^−03^				
			iii	A/G	0.1225	0.1343	0.90 (0.73, 1.03)	7.60 × 10^−02^				
4q34	rs997779	*GPM6A*	i	C/A	0.1324	0.1019	1.35 (1.12, 1.62)	1.50 × 10^−03^	1.17 (1.08, 1.26)	4.48 × 10^−02^	0.07	69.38
			ii	C/A	0.1216	0.1080	1.15 (1.00, 1.31)	4.54 × 10^−02^				
			iii	C/A	0.1250	0.1113	1.17 (1.08, 1.26)	1.32 × 10^−02^				
5q34	rs2431697	*MIR146A*	i	G/A	0.1391	0.1846	0.71 (0.61, 0.84)	3.76 × 10^−05^	0.69 (0.65, 0.75)	1.15 × 10^−22^	0.68	0
			ii	G/A	0.1410	0.1706	0.80 (0.71, 0.90)	1.49 × 10^−04^				
			iii	G/A	0.1250	0.1722	0.69 (0.58, 0.82)	3.95 × 10^−19^				
5q34	rs1862390	*MIR146A*	i	G/A	0.3552	0.3934	0.85 (0.75, 0.96)	8.30 × 10^−03^	0.87 (0.81, 0.94)	1.37 × 10^−04^	0.59	0
			ii	G/A	0.3497	0.3745	0.90 (0.82, 0.98)	1.59 × 10^−02^				
			iii	G/A	0.4833	0.4363	1.20 (0.70, 2.07)	5.30 × 10^−01^				
11p13	rs2732547	*CD44*	i	A/G	0.2156	0.2560	0.80 (0.70, 0.92)	1.47 × 10^−03^	0.82 (0.77, 0.87)	1.55 × 10^−11^	0.82	0
			ii	A/G	0.2170	0.2479	0.84 (0.76, 0.93)	6.50 × 10^−04^				
			iii	A/G	0.2075	0.2436	0.81 (0.67, 0.83)	2.56 × 10^−09^				
11p13	rs1559759	*CD44*	i	A/C	0.3518	0.3145	1.18 (1.05, 1.34)	8.04 × 10^−03^	1.08 (1.03, 1.14)	4.57 × 10^−02^	0.14	55.20
			ii	A/C	0.3515	0.3266	1.12 (1.02, 1.22)	1.42 × 10^−02^				
			iii	A/C	0.3386	0.3350	1.02 (0.93, 1.11)	2.52 × 10^−01^				
15q24.1	rs881536	*ULK3*	i	A/C	0.4431	0.3925	1.23 (1.09, 1.39)	5.94 × 10^−04^	1.16 (1.07, 1.23)	5.78 × 10^−03^	0.15	51.34
			ii	A/C	0.4187	0.3942	1.11 (1.02, 1.20)	1.99 × 10^−02^				
15q26.2	rs2535483	*SPATA8*	i	A/G	0.3356	0.2871	1.25 (1.11, 1.42)	4.56 × 10^−04^	1.06 (0.96, 1.15)	7.36 × 10^−01^	0	94.28
			ii	A/G	0.2825	0.3047	0.90 (0.82, 0.98)	2.26 × 10^−02^				
16q24.1	rs2934498	*IRF8*	i	G/A	0.3789	0.3380	1.20 (1.06, 1.35)	4.29 × 10^−03^	1.25 (1.16, 1.34)	4.97 × 10^−09^	0.40	0
			ii	G/A	0.3915	0.3353	1.28 (1.17, 1.39)	3.31 × 10^−07^				

*Stage i: genotyping in 2,252 individuals by Illumina Human 610-Quad BeadChips; stage ii: genotyping in 4,410 individuals by the Sequenom MassArray system; stage iii: genotyping in 7,345 individuals by the Sequenom MassArray system. ^a^ *P-*values from the Cochran-Armitage trend test. ^b^ *P-*values from fix or random joint analysis (see Subjects and Methods).Chr, chromosome; MAF, minor allele frequency; OR, odds ratio.

### Association results of GWAS, validation stages and combined all samples

We then genotyped these 12 SNPs with suggestive association evidence in the replication 2. The association analysis in replication 2 revealed consistent association with SLE for 7 of 12 SNPs with the GWAS and replication 1 stages. When we combined the genotypic data from the GWAS and two independent replication cohorts, an additional three SNPs within three known SLE susceptibility loci reach genome-wide levels of significance: *miR-146a* (rs2431697, OR = 0.69, *P*_meta_ = 1.15 × 10^−22^), *CD44* (rs2732547, OR = 0.82, *P*_meta_ = 1.55 × 10^−11^), and *TMEM39A* (rs12494314, OR = 0.84, *P*_meta_ = 1.01 × 10^−09^) (Table 2).

## Discussion

In the present study, six SNPs in five known SLE susceptibility loci showed evidence of highly significant association evidence in combined analysis: *TNFSF4* (rs1418190, rs4916219), *IRF8* (rs2934498), *miR-146a* (rs2431697), *CD44* (rs2732547), and *TMEM39A* (rs12494314). At 1q25, we identified two new susceptibility SNPs (rs1418190, rs4916219) in *TNFSF4*, which are independent from two reported SNPs (rs1234315, rs2205960) [11] through logistic regression analysis based on GWAS data. In addition, LD analysis showed that there is no evidence of strong LD among them (D’ <0.22, *r*^2^ <0.02) (Additional file 2: Table S2). *TNFSF4* (tumor necrosis factor (ligand) superfamily, member 4) (also known as *OX40L*), belongs to the TNF ligand family, encodes a cytokine that is involved in T cell antigen-presenting cell interactions [26]. The interaction between *OX40L* and its receptor (*OX40*) has a dual effect by delivering a strong co-stimulatory signal to activated effector T-cells and enhances both Th1 and Th2 responses [27], and inhibiting the generation and function of IL-10-producing CD4+ type 1 regulatory T cells [28]. Furthermore, signaling through *TNFSF4* is shown to induce B cell activation and differentiation [29], which results in the production of auto-antibodies and immune complexes.

The genetic polymorphisms (rs2280381, rs11644034) in *IRF8* (interferon regulatory factor 8) have been confirmed to confer susceptibility to lupus in European populations [30,31]. Although these European SNPs were observed in our GWAS data, no association was discovered with the Chinese population (rs2280381, *P* = 0.13; rs11644034, *P* = 0.26). In the present study, we identified a new SNP rs2934498 near rs2280381 (D’ = 0.325, *r*^2^ = 0.008) and rs11644034 (D’ = 0.984, *r*^2^ = 0.049), indicating that *IRF8* is also susceptible to SLE in the Chinese population. Previous studies have shown that *IRF8* plays a critical role in regulating the differentiation of myeloid and B-cells and can be induced by interferon-γ in macrophages and antigen stimulation within T cells [32,33]. Interestingly, the overexpression of genes induced by type I INF has been widely reported in SLE and other autoimmune diseases [34-36].

As for 5q34, we confirmed an associated variant (rs2431697) between the *PTTG1* (pituitary tumor-transforming 1) and the *miR-146a* (microRNA 146a) genes, which has been identified in previous study in women of European ancestry with SLE. *miR-146a* is a microRNA (miRNA) that has been shown to be involved in both the regulation of innate and adaptive immune system and tumor progression [37]. Recently, gene expression analysis has revealed that this SNP is not associated with *PTTG1* expression levels, but with the *miR-146a*, where the risk allele correlates with downregulation of the *miR-146a*, potentially important in SLE etiology [38].

*CD44* (CD44 molecule) encodes a transmembrane receptor, which is important for lymphocyte activation, recirculation and homing, apoptosis, hematopoiesis, and tumor metastasis [39,40]. Several studies have identified SPP1, a ligand for CD44, as an SLE risk locus involved in IFN pathways [41,42]. Dysregulation of the IFN system plays critical roles in the pathogenesis of SLE and other closely related autoimmune phenotypes [36]. T cells from SLE patients also display increased and abnormal distribution of *CD44* [43], meanwhile, the overexpression of *CD44v3* and *CD44v6* isoforms in T cells was also observed in the blood of SLE patients, and correlated with disease activity [44]. Several studies have identified multiple variants (rs2732552, rs507230) within or nearby *CD44* that are associated with SLE in various populations [45,46]. However, rs2732552 was not covered in our GWAS data and rs507230 did not show any association with SLE in our GWAS data (*P* = 0.84). In this study, we determined that another SNP rs2732547 near rs507230 (D’ = 0.259, *r*^2^ = 0.067), indicating that *CD44* is also associated with SLE in the Chinese population.

At 3q13.33, a coding SNP (rs1132200) in *TMEM39A* has been reported to be associated with both SLE [31] and multiple sclerosis [47]. However, rs1132200 was not covered in our GWAS data. In the current study, we discovered another SLE-susceptibility SNP (rs12494314) in this region. To date, very few biological data on *TMEM39A* have been published to provide evidence of its relevance to SLE [31,47].

To investigate the potential molecular mechanism of six significant genetic variants associated with SLE we performed bioinformatics analysis by inferring the relevant biological function from the diverse genomic data and computational prediction. We first utilized ENCODE chromatin state data (three tie 1 cell type: GM12878, K562, H1 human embryonic stem cells) and comprehensive annotations [48] to inspect whether those single nucleotide variants (SNVs) covered by promoter/enhancer/insulator markers (H3K4me1, H3K27ac, p300, CTCF, DNase I hypersensitive site) which indicate the active transcriptional signals. We found rs2431697 (*miR-146a*), as the leading SNV in GWAS, obtained strong enhancer signals (H3K4me1 and H3K27ac). It is noticeable that rs2431697 (*miR-146a*) is located in a conserved region by mapping the variant to GERP++ conservation elements [49]. Another leading SNV rs2732547 (*CD44*) is also marked by p300 and H3K4me1 signals. Therefore, we speculated that those two genetic variants are associated with SLE by affecting the transcription factors binding in their located enhancers. In order to find the affected transcription factors and quantitatively measure the change of binding affinity, we used motifs of different transcription factors from Jasper [50] to scan the surrounding sequences of the above two genetic variants in a different allele state. We then calculated the difference in transcription factor binding score and prioritized them based on the respective *P*-value. For rs2431697 (*miR-146a*), we predicted that the binding of transcriptional repressor protein YY1 (YY1 gene) may be disrupted in the corresponding variant region of minor allele G. Importantly, we observed a very significant binding affinity change for androgen receptor (NR3C4) in the rs2732547 (*CD44*) located region for a different allele. The minor allele A will significantly reduce the binding activity of the androgen receptor and then may result in the development of SLE. The androgen is important in clinical treatment of SLE [51] and the correlation between the androgen receptor and SLE has been investigated by different studies [52,53]. For other associated SNVs, we did not detect sufficient evidence and signals at the transcriptional level.

## Conclusions

In conclusion, this study confirmed five previous reported SLE susceptibility loci in Han Chinese population, such as *TNFSF4*, *IRF8*, *miR-146a*, *CD44* and *TMEM39A*. These findings not only provide novel insights into the genetic architecture of SLE but also might highlight the contribution of multiple variants of modest effect.

This study was approved by the ethics committee of The First Affiliated Hospital of Anhui Medical University and conducted according to the principles of the Declaration of Helsinki.

Patient consent was obtained.

## Additional files

Additional file 1: Table S1. Summary of the association results for 34 SNPs in the GWAS and replication 1 and combined studies. Additional file 2: Table S2. Conditional analysis between two new susceptibility SNPs (rs1418190, rs4916219) and two reported SNPs (rs1234315, rs2205960) in GWAS samples. Additional file 3: Table S3. Haplotypic association for markers in *TNFSF4* locus with the risk of SLE.

## Abbreviations

ACR: American College of Rheumatology
AHMU: Anhui Medical University
ANA: antinuclear antibody
Chr: chromosome
GWAS: genome-wide association studies
HWE: Hardy-Weinberg equilibrium
INF: interferon
LD: linkage disequilibrium
MAF: minor allele frequency
OR: odds ratio
SLE: systemic lupus erythematosus
SNP: single nucleotide polymorphism
SNV: single nucleotide variant
