# Supplementary material for: Association analyses confirm five susceptibility loci for systemic lupus erythematosus in the Han Chinese population
Source: Arthritis Res Ther. 2015 Mar 28;17(1):85. doi: 10.1186/s13075-015-0602-9 (PMC4404072; doi:10.1186/s13075-015-0602-9)
Supplement: Additional file 1: Table S1. — Summary of the association results for 34 SNPs in the GWAS and replication 1 and combined studies. [file 13075_2015_602_MOESM1_ESM.docx]

**Supplementary Table 1. Summary of the association results for 34 SNPs in the GWAS and replication 1 and combined studies.**

| Chr | SNP | BP | Gene | Allele | GWAS | | | |  | Replication 1 | | | |  | Meta-analysis | | | | I^2^ |
| --- | --- | --- | --- | --- | --- | --- | --- | --- | --- | --- | --- | --- | --- | --- | --- | --- | --- | --- | --- |
|  |  |  |  |  | 1,047 cases, 1,205 controls | | | |  | 2,202 cases, 2,208 controls | | | |  | 3,249 cases, 3,413 controls | | | |  |
|  |  |  |  |  | MAF | | OR (95%CI) | *P^a^* |  | MAF | | OR (95%CI) | *P^a^* |  | MAF | | OR (95%CI) | *P^b^* |  |
|  |  |  |  |  | Case | Control |  |  |  | Case | Control |  |  |  | Case | Control |  |  |  |
| 4p15.33 | rs10028728 | 13692050 | *LOC152742* | A/G | 0.1592 | 0.1270 | 1.30 (1.10-1.54) | 2.01E-03 |  | 0.1356 | 0.1381 | 0.98 (0.87-1.11) | 7.33E-01 |  | 0.1433 | 0.1341 | 1.12 (0.98-1.19) | 4.17E-01 | 86.17 |
| 5q21 | rs10062255 | 173196477 | *LOC729170* | A/C | 0.4312 | 0.3805 | 1.23 (1.10-1.39) | 5.49E-04 |  | 0.3947 | 0.4097 | 0.94 (0.86-1.02) | 1.52E-01 |  | 0.4065 | 0.3994 | 1.07 (0.96-1.10) | 6.06E-01 | 92.47 |
| 2q22.3 | rs10496992 | 146382727 | *LOC728773* | G/A | 0.3394 | 0.3880 | 0.81 (0.72-0.92) | 7.36E-04 |  | 0.3666 | 0.3641 | 1.01 (0.93-1.10) | 8.05E-01 |  | 0.3577 | 0.3724 | 0.91 (0.87-1.01) | 3.93E-01 | 88.10 |
| 12q14.3 | rs10878883 | 67661803 | *CPM* | A/G | 0.4312 | 0.4876 | 0.80 (0.71-0.90) | 1.54E-04 |  | 0.4510 | 0.4681 | 0.93 (0.86-1.02) | 1.10E-01 |  | 0.4445 | 0.4749 | 0.87 (0.83-0.95) | 7.41E-02 | 78.51 |
| 12p11.22 | rs11050116 | 29242788 | *MLSTD1* | C/A | 0.2438 | 0.2087 | 1.22 (1.06-1.41) | 4.95E-03 |  | 0.2015 | 0.2179 | 0.91 (0.82-1.01) | 6.11E-02 |  | 0.2154 | 0.2146 | 1.05 (0.92-1.09) | 7.52E-01 | 91.16 |
| 4q34.3 | rs11132111 | 183244670 | *MGC45800* | A/G | 0.3719 | 0.3270 | 1.22 (1.08-1.38) | 1.60E-03 |  | 0.3406 | 0.3402 | 1.00 (0.92-1.10) | 9.76E-01 |  | 0.3507 | 0.3354 | 1.10 (1.00-1.15) | 3.31E-01 | 84.45 |
| 11p11.2 | rs11600829 | 44582069 | *CD82* | G/A | 0.4025 | 0.3488 | 1.26 (1.11-1.42) | 2.07E-04 |  | 0.3576 | 0.3673 | 0.96 (0.88-1.05) | 3.48E-01 |  | 0.3723 | 0.3608 | 1.09 (0.98-1.13) | 5.06E-01 | 92.07 |
| 5q11.2-q13 | rs12515008 | 63061975 | *HTR1A* | G/A | 0.3987 | 0.3490 | 1.24 (1.10-1.40) | 5.80E-04 |  | 0.3574 | 0.3639 | 0.97 (0.89-1.06) | 5.29E-01 |  | 0.371 | 0.3588 | 1.09 (0.98-1.13) | 4.68E-01 | 90.07 |
| 18p11.32 | rs12605083 | 5594170 | *EPB41L3* | C/A | 0.1431 | 0.1175 | 1.25 (1.05-1.49) | 1.09E-02 |  | 0.1086 | 0.1124 | 0.96 (0.84-1.10) | 5.74E-01 |  | 0.1198 | 0.1143 | 1.09 (0.95-1.17) | 5.13E-01 | 82.31 |
| 2q33 | rs1429890 | 209216592 | *PTH2R* | A/G | 0.4575 | 0.4967 | 0.85 (0.76-0.96) | 8.60E-03 |  | 0.4745 | 0.4727 | 1.01 (0.93-1.10) | 8.66E-01 |  | 0.469 | 0.4813 | 0.93 (0.89-1.02) | 3.94E-01 | 79.85 |
| 3p25.2 | rs1466835 | 12554944 | *TSEN2* | A/C | 0.1372 | 0.1087 | 1.30 (1.09-1.56) | 3.59E-03 |  | 0.1254 | 0.1210 | 1.04 (0.92-1.18) | 5.34E-01 |  | 0.1293 | 0.1166 | 1.16 (1.01-1.25) | 1.97E-01 | 75.00 |
| 6q21 | rs1508371 | 106351673 | *PRDM1* | A/G | 0.2148 | 0.1830 | 1.22 (1.06-1.42) | 7.48E-03 |  | 0.1810 | 0.1800 | 1.01 (0.90-1.12) | 8.98E-01 |  | 0.192 | 0.181 | 1.10 (0.99-1.17) | 3.09E-01 | 76.48 |
| 2q22.3 | rs1567989 | 146397788 | *PABPC1P2* | A/C | 0.4133 | 0.4573 | 0.84 (0.74-0.94) | 3.04E-03 |  | 0.4421 | 0.4410 | 1.01 (0.92-1.09) | 9.13E-01 |  | 0.4327 | 0.4466 | 0.92 (0.88-1.01) | 3.76E-01 | 83.86 |
| 3p26.1-p25.1 | rs17047582 | 7566405 | *GRM7* | G/A | 0.1549 | 0.1187 | 1.36 (1.15-1.62) | 4.00E-04 |  | 0.1354 | 0.1336 | 1.02 (0.90-1.15) | 8.06E-01 |  | 0.1417 | 0.1283 | 1.17 (1.02-1.24) | 2.86E-01 | 86.48 |
| 2q32-q34 | rs1882396 | 191424257 | *GLS* | A/C | 0.2275 | 0.2643 | 0.82 (0.72-0.94) | 4.33E-03 |  | 0.2610 | 0.2769 | 0.92 (0.84-1.02) | 9.39E-02 |  | 0.2502 | 0.2724 | 0.88 (0.82-0.96) | 2.70E-02 | 48.69 |
| 6q22 | rs195078 | 119698645 | *MAN1A1* | A/C | 0.3279 | 0.3664 | 0.84 (0.75-0.95) | 6.89E-03 |  | 0.3649 | 0.3544 | 1.05 (0.96-1.14) | 3.07E-01 |  | 0.3529 | 0.3586 | 0.94 (0.91-1.05) | 5.94E-01 | 87.22 |
| 12q21 | rs1982909 | 79688148 | *LIN7A* | A/G | 0.4269 | 0.4801 | 0.81 (0.72-0.91) | 3.49E-04 |  | 0.4431 | 0.4571 | 0.95 (0.87-1.03) | 1.91E-01 |  | 0.4378 | 0.4651 | 0.88 (0.84-0.96) | 1.02E-01 | 78.40 |
| 22q13.1 | rs2235148 | 33994941 | *HMG2L1* | A/G | 0.4019 | 0.4435 | 0.84 (0.75-0.95) | 4.92E-03 |  | 0.4129 | 0.4306 | 0.93 (0.85-1.01) | 9.36E-02 |  | 0.4093 | 0.4351 | 0.89 (0.84-0.96) | 1.92E-02 | 41.85 |
| 13q14.2 | rs2247119 | 48985143 | *PHF11* | G/A | 0.4460 | 0.4004 | 1.21 (1.07-1.36) | 2.01E-03 |  | 0.4270 | 0.4063 | 1.09 (1.00-1.19) | 5.07E-02 |  | 0.4333 | 0.4041 | 1.14 (1.05-1.21) | 9.79E-03 | 44.71 |
| 22q12.1 | rs2283817 | 25253155 | *TPST2* | G/A | 0.4202 | 0.4623 | 0.84 (0.75-0.95) | 4.83E-03 |  | 0.4536 | 0.4297 | 1.10 (1.00-1.22) | 5.61E-02 |  | 0.4427 | 0.4456 | 0.97 (0.92-1.07) | 7.95E-01 | 91.28 |
| 15q23-q25 | rs2305668 | 72929814 | *SCAMP2* | C/A | 0.2165 | 0.2492 | 0.83 (0.72-0.96) | 9.92E-03 |  | 0.2351 | 0.2420 | 0.96 (0.87-1.06) | 4.46E-01 |  | 0.229 | 0.2445 | 0.90 (0.85-0.99) | 1.59E-01 | 64.16 |
| 5q32 | rs4259160 | 147537383 | *SPINK5L2* | A/G | 0.4441 | 0.4851 | 0.85 (0.75-0.95) | 5.96E-03 |  | 0.4594 | 0.4543 | 1.02 (0.90-1.16) | 7.51E-01 |  | 0.4503 | 0.4685 | 0.93 (0.85-1.01) | 4.27E-01 | 77.23 |
| 2p14 | rs4671866 | 68075181 | *LOC402076* | A/G | 0.2675 | 0.2320 | 1.21 (1.06-1.39) | 5.94E-03 |  | 0.2402 | 0.2413 | 0.99 (0.90-1.10) | 9.07E-01 |  | 0.2491 | 0.2379 | 1.09 (0.98-1.15) | 3.77E-01 | 80.9 |
| 6q27 | rs4710140 | 167252265 | *RNASET2* | A/G | 0.1252 | 0.0966 | 1.34 (1.11-1.62) | 2.19E-03 |  | 0.1048 | 0.1014 | 1.04 (0.90-1.19) | 5.96E-01 |  | 0.1114 | 0.09965 | 1.17 (1.01-1.27) | 2.15E-01 | 78.12 |
| 16p13.3 | rs4786664 | 5289136 | *RBFOX1* | A/C | 0.3035 | 0.3419 | 0.84 (0.74-0.95) | 6.08E-03 |  | 0.3434 | 0.3359 | 1.03 (0.95-1.13) | 4.62E-01 |  | 0.3304 | 0.3381 | 0.94 (0.90-1.04) | 5.26E-01 | 85.93 |
| 19q12 | rs4805420 | 34665351 | *LOC284395* | A/G | 0.4283 | 0.4793 | 0.81 (0.72-0.92) | 6.17E-04 |  | 0.4658 | 0.4647 | 1.00 (0.92-1.09) | 9.22E-01 |  | 0.4536 | 0.4701 | 0.91 (0.87-1.00) | 3.52E-01 | 87.48 |
| 1q42.12 | rs597429 | 224850811 | *C1orf95* | A/G | 0.3633 | 0.4058 | 0.84 (0.74-0.94) | 3.47E-03 |  | 0.4063 | 0.3852 | 1.09 (1.00-1.19) | 4.42E-02 |  | 0.3923 | 0.3925 | 0.96 (0.93-1.07) | 7.51E-01 | 92.04 |
| 4p16.1 | rs6847759 | 6644018 | *MAN2B2* | G/A | 0.4015 | 0.4407 | 0.85 (0.76-0.96) | 8.02E-03 |  | 0.4340 | 0.4400 | 0.98 (0.90-1.06) | 5.71E-01 |  | 0.4234 | 0.4403 | 0.92 (0.87-1.00) | 2.04E-01 | 70.04 |
| 1p36.2 | rs7417097 | 8916454 | *CA6* | G/A | 0.3287 | 0.3800 | 0.80 (0.71-0.90) | 3.41E-04 |  | 0.3563 | 0.3544 | 1.01 (0.92-1.10) | 8.54E-01 |  | 0.3473 | 0.3637 | 0.90 (0.87-1.00) | 3.66E-01 | 88.93 |
| 6p25.1 | rs7760489 | 4662212 | *CDYL* | G/A | 0.2930 | 0.2475 | 1.26 (1.10-1.44) | 5.92E-04 |  | 0.2730 | 0.2780 | 0.97 (0.89-1.07) | 5.99E-01 |  | 0.2795 | 0.2672 | 1.10 (0.99-1.15) | 4.44E-01 | 89.63 |
| 6q23.3 | rs7775153 | 137827982 | *OLIG3* | A/G | 0.1816 | 0.2249 | 0.77 (0.66-0.89) | 3.35E-04 |  | 0.2200 | 0.2092 | 1.07 (0.96-1.18) | 2.19E-01 |  | 0.2076 | 0.2149 | 0.91 (0.88-1.04) | 5.53E-01 | 92.40 |
| 7p14.3 | rs7785659 | 32427540 | *SLC25A5P5* | A/G | 0.1726 | 0.1313 | 1.38 (1.17-1.63) | 1.15E-04 |  | 0.1549 | 0.1449 | 1.08 (0.96-1.22) | 1.89E-01 |  | 0.1607 | 0.14 | 1.21 (1.07-1.29) | 1.09E-01 | 81.85 |
| 13q14.3 | rs796906 | 49646285 | *FAM10A4* | A/G | 0.3542 | 0.3971 | 0.83 (0.74-0.94) | 3.06E-03 |  | 0.3957 | 0.3779 | 1.08 (0.99-1.18) | 8.91E-02 |  | 0.3821 | 0.3846 | 0.95 (0.92-1.06) | 7.00E-01 | 91.40 |
| 9q21.33 | rs943855 | 89460147 | *DAPK1* | G/A | 0.4025 | 0.3485 | 1.26 (1.12-1.42) | 1.91E-04 |  | 0.3775 | 0.3598 | 1.08 (0.99-1.18) | 8.73E-02 |  | 0.3857 | 0.3559 | 1.16 (1.06-1.22) | 5.56E-02 | 75.67 |

CI, confidence interval; MAF, minor allele frequency; OR, odds ratio; SNP, single-nucleotide polymorphism. ^a^*P* values from the Cochran-Armitage trend test. ^b^*P* values from fix or random joint analysis (see Subjects and Methods).
