# Supplementary material for: Association analyses confirm five susceptibility loci for systemic lupus erythematosus in the Han Chinese population
Source: Arthritis Res Ther. 2015 Mar 28;17(1):85. doi: 10.1186/s13075-015-0602-9 (PMC4404072; doi:10.1186/s13075-015-0602-9)
Supplement: Additional file 2: Table S2. — Conditional analysis between two new susceptibility SNPs (rs1418190, rs4916219) and two reported SNPs (rs1234315, rs2205960) in GWAS samples. [file 13075_2015_602_MOESM2_ESM.docx]

**Supplementary Table 2. Conditional analysis between two new susceptibility** **SNPs (rs1418190, rs4916219) and two reported SNPs (rs1234315, rs2205960) in GWAS samples.**

| Chr | SNP | Gene | Allele | GWAS stage | | | |  | Logistic condition | | | LD | |
| --- | --- | --- | --- | --- | --- | --- | --- | --- | --- | --- | --- | --- | --- |
|  |  |  |  | MAF | | *P* valve | OR (95% CI) |  | Condition | *P* value | OR (95% CI) | D’ | r^2^ |
|  |  |  |  | Cases | Controls |  |  |  |  |  |  |  |  |
| 1q25 | **rs1418190** | *TNFSF4* | G/A | 0.3089 | 0.3579 | 5.26×10^-04^ | 0.80 (0.71-0.91) |  | rs1234315 | 2.59×10^-05^ | 0.76 (0.67-0.86) | 0.168 | 0.017 |
|  |  |  |  |  |  |  |  |  | rs2205960 | 6.56×10^-04^ | 0.80 (0.71-0.91) | 0.033 | 0.000 |
|  |  |  |  |  |  |  |  |  | **rs4916219** | 1.64×10^-01^ | 0.85 (0.67-1.07) | 0.926 | 0.715 |
| 1q25 | **rs4916219** | *TNFSF4* | A/G | 0.2722 | 0.3166 | 1.15×10^-03^ | 0.81 (0.71-0.92) |  | rs1234315 | 3.93×10^-05^ | 0.76 (0.66-0.86) | 0.214 | 0.023 |
|  |  |  |  |  |  |  |  |  | rs2205960 | 8.42×10^-04^ | 0.80 (0.70-0.91) | 0.007 | 0.000 |
|  |  |  |  |  |  |  |  |  | **rs1418190** | 5.73×10^-01^ | 0.93 (0.73-1.19) | 0.926 | 0.715 |
| 1q25 | rs1234315 | *TNFSF4* | A/G | 0.5000 | 0.4253 | 5.31×10^-07^ | 1.35 (1.20-1.52) |  | rs2205960 | 1.37×10^-01^ | 1.13 (0.96-1.33) | 0.960 | 0.457 |
|  |  |  |  |  |  |  |  |  | **rs1418190** | 1.37×10^-08^ | 1.40 (1.24-1.33) | 0.168 | 0.017 |
|  |  |  |  |  |  |  |  |  | **rs4916219** | 3.05×10^-08^ | 1.40 (1.25-1.58) | 0.214 | 0.023 |
| 1q25 | rs2205960 | *TNFSF4* | A/C | 0.3394 | 0.2602 | 6.55×10^-09^ | 1.46 (1.29-1.66) |  | rs1234315 | 1.48×10^-03^ | 1.33 (1.11-1.58) | 0.960 | 0.457 |
|  |  |  |  |  |  |  |  |  | **rs1418190** | 1.60×10^-08^ | 1.45 (1.27-1.65) | 0.033 | 0.000 |
|  |  |  |  |  |  |  |  |  | **rs4916219** | 9.28×10^-09^ | 1.46 (1.28-1.66) | 0.007 | 0.000 |

MAF, minor allele frequency.

Allele, minor allele/major allele.
