# Supplementary material for: Association analyses confirm five susceptibility loci for systemic lupus erythematosus in the Han Chinese population
Source: Arthritis Res Ther. 2015 Mar 28;17(1):85. doi: 10.1186/s13075-015-0602-9 (PMC4404072; doi:10.1186/s13075-015-0602-9)
Supplement: Additional file 3: Table S3. — Haplotypic association for markers in TNFSF4 locus with the risk of SLE. [file 13075_2015_602_MOESM3_ESM.docx]

**Supplementary Table 3. Haplotypic association for markers in *TNFSF4* locus with the risk of SLE**

| Haplotype | rs1234315 | rs2205960 | **rs1418190** | **rs4916219** | GWAS stage | | | |
| --- | --- | --- | --- | --- | --- | --- | --- | --- |
|  |  |  |  |  | Freq_ cases | Freq_ controls | OR (95%CI) | *P* |
| 1 | G | C | A | G | 0.376 | 0.383 | - | - |
| 2 | G | C | G | A | 0.086 | 0.140 | 0.62 (0.48-0.80) | 3.67×10^-08^ |
| 3 | A | A | A | G | 0.219 | 0.168 | 1.31 (1.08-1.58) | 1.42×10^-07^ |

Haplotype 1 was set as reference. Freq_cases/Freq_controls: the haplotype frequency in cases/controls; *P*: the association *P* values for each haplotype in the Han Chinese population; OR: odds ratio; 95% CI: 95% confidence interval.
